# Leveraging Artificial Intelligence to Expedite Antibody Design and Enhance Antibody–Antigen Interactions

**DOI:** 10.3390/bioengineering11020185

**Published:** 2024-02-15

**Authors:** Doo Nam Kim, Andrew D. McNaughton, Neeraj Kumar

**Affiliations:** Pacific Northwest National Laboratory, 902 Battelle Blvd., Richland, WA 99352, USA; doonam.kim@pnnl.gov (D.N.K.); andrew.mcnaughton@pnnl.gov (A.D.M.)

**Keywords:** antibody, artificial intelligence, computer-aided drug discovery, computational modeling and simulations, deep learning, protein–protein interface, Rosetta, therapeutic design

## Abstract

This perspective sheds light on the transformative impact of recent computational advancements in the field of protein therapeutics, with a particular focus on the design and development of antibodies. Cutting-edge computational methods have revolutionized our understanding of protein–protein interactions (PPIs), enhancing the efficacy of protein therapeutics in preclinical and clinical settings. Central to these advancements is the application of machine learning and deep learning, which offers unprecedented insights into the intricate mechanisms of PPIs and facilitates precise control over protein functions. Despite these advancements, the complex structural nuances of antibodies pose ongoing challenges in their design and optimization. Our review provides a comprehensive exploration of the latest deep learning approaches, including language models and diffusion techniques, and their role in surmounting these challenges. We also present a critical analysis of these methods, offering insights to drive further progress in this rapidly evolving field. The paper includes practical recommendations for the application of these computational techniques, supplemented with independent benchmark studies. These studies focus on key performance metrics such as accuracy and the ease of program execution, providing a valuable resource for researchers engaged in antibody design and development. Through this detailed perspective, we aim to contribute to the advancement of antibody design, equipping researchers with the tools and knowledge to navigate the complexities of this field.

## 1. Introduction

**Figure 1:**
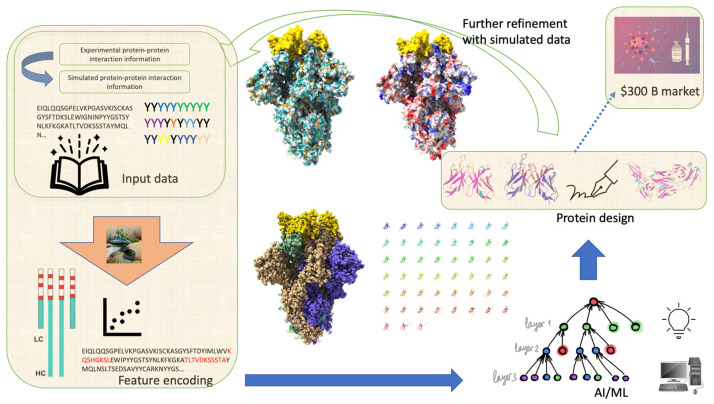
Generalized schematic of DL approaches for Ab sequence design. The three large middle molecules are SARS Corona virus 2 (SARS-CoV-2) spikes (PDB ID: 7kkl). Three bound nanobody (Nb) molecules are depicted with yellow caps. Hydrophobicity potential ranges from cyan for hydrophilic through white to goldenrod for hydrophobic. Electrostatic potential ranges from red for negative potential through white to blue for positive potential. The rainbow-colored array of molecules is Nb, a single-chain Ab.

Living organisms can contain foreign substances, such as viruses or toxins, which are known as antigens (Ags). The presence of Ags triggers immune responses in the body, including the production of antibodies (Abs). The interaction between Abs and Ags has become increasingly important due to the expanding use of Ab-based therapeutics and diagnostics. With over 100 monoclonal Abs (mAbs) approved by the US Food and Drug Administration (FDA) [1,2], these approaches tend to exhibit lower toxicity and higher specificity control compared to traditional small molecule-based therapeutics. Consequently, the global therapeutic mAb market is anticipated to reach USD 300 billion by 2025 [3]. However, optimizing Abs experimentally is a laborious process because of the low-throughput nature of Ab screening in mammalian cells. For instance, yeast and phage display only cover between 10^6^ and 10^10^ Ab sequences [4]; while most Ab sequence screenings in industry often exceed 10^11^. Therefore, significant progress has been made in the application of deep learning (DL) towards Ab discovery, as reviewed in multiple studies [4,5,6,7,8]. These advancements encompass the modeling and prediction of Ab–target binding patterns for the identification of binding sequences [9], paratope prediction [10], complementarity-determining region (CDR) loop structure prediction [11], and target specificity. In this work, we present a review to discuss recent developments in DL approaches for Ab sequence design (Figure 1). A promising trend in the field of DL is the widespread practice of researchers publicly sharing their code and publicly shared Ab affinity datasets [12,13]. This collaborative approach, apart from a few commercial entities, has facilitated synergy among disciplines. It is hoped that these collaborative efforts will continue to strengthen and eventually reach the level of successful collaborative groups such as the *Rosetta* community [14].

### 1.1. Historical Perspective and Rise of Deep Learning in the Biomedical Field

DL serves as a critical universal approximator capable of generalizing complex, nonlinear phenomena [15]. As a result, DL has been actively implemented in various fields related to protein design and small-molecule drug design. These advancements in DL approaches include the study of non-coding RNA–protein interactions [16], compound–protein interactions [17], the annotation of protein space [18] and gene ontology (GO) [19], and three-dimensional (3D) coordinates of drug-like molecules [20]. Likewise, DL methods facilitate Ab development. For example, a combination of a convolutional neural network (CNN) and a recurrent neural network (RNN) including a conditional random field [21] was used to predict the signal peptide cleavage site of recombinant mAbs to reduce product heterogeneity issues [22]. Most importantly, DL approaches often need to be integrated with other physics-based modeling and simulation methods. For instance, many modern DL protein modeling methods, including *AlphaFold* version 1 [23], typically refine the final structure using *Rosetta*’s *FastRelax* [24]. However, a more precise DL-based amino acid (AA) packer would be ideal. In this context, utilizing *AttnPacker* [25] appears to be the optimal choice, as it reduces the inference time by over 100× compared to other DL-based methods, such as *DLPacker* [26], and physics-based method *FastRelax*.

When predicting protein–protein interactions (PPIs) or PPI complex structures, a DL-based tool called AF2Complex [27] is currently known to produce the most accurate results according to the DockQ score [28]. We discovered that using AlphaFold-Multimer [29] was simpler in terms of execution and analysis (see Supplementary Note S1 for details), which provides not only accurate outcomes but also produces a readily understandable iPTM report. On the other hand, the most recent docking and design model specifically tailored for Ab–Ag interactions is the Hierarchical Structure Refinement Network. It has improved Ab docking success rates by 50%, outperforming other sequence-based and structure-based models [30]. As demonstrated with the transformer architecture [31], attention-based networks are powerful methods to capture interactions between input data and often result in better accuracy [12]. Since it is believed that most epitopes are somewhat discontinuous [32], it is critical to understand recent Ab–Ag interface-based DL models [12,15,33] that have adopted attention-based architectures. Ideally, attention-based methods that consider both the sequence and structural context would be more suitable for sequence generation [34]. The superior accuracy of DL over non-DL methods is often evident, as seen in immune status classification based on immune repertoire sequences [35] and Fv structure prediction [12]. 

Enhancements in Ab affinity often result from combined AA substitutions rather than individual site mutations [13]. Surprisingly, many affinity-boosting mutations are found in areas that do not directly interact with the Ag. These mutations can lead to voids, misaligned polar AA, and spatial conflicts, making them difficult to predict [36]. Therefore, utilizing DL architectures for Ab design [12] might present advantages over traditional computational methods [37]. Despite this, we concur that DL-based PPI modeling methods may not always be the optimal choice, particularly due to the insufficient availability of training data or when training is not robust. For instance, Dequeker et al. demonstrated that their comprehensive cross-docking approach surpassed a sequence-based DL method [38].

### 1.2. Sequence-Based and Structure-Based Approaches with Implications for Antibody Design

Ab design methodologies can be classified into sequence-based or structure-based methods, contingent upon the types of input and output data. In this review, we delve into each of these categories with relevant examples and evaluations. In this subsection, we provide a summary of each category. The development of Ab-based therapeutics typically spans several years, and enhancing therapeutic efficacy can result in substantial cost savings. In order to assess therapeutic efficacy at an early stage, a number of prediction methods based on DL have been developed to predict Ab affinity [13,15,39]. As we analyzed, it is clear that most of these DL-assisted Ab modeling approaches only require protein sequence data as input. This is an ideal approach because it allows for quick implementation of the model [40], and, most importantly, Ab sequence data can be produced on a much larger scale, significantly lowering costs compared to generating structural information about Abs [41]. Similar to the situation present in general protein data, there is a huge amount of sequence data available (for example, metagenomic sequences surpass 1.6 billion [42], while the Protein Data Bank (PDB) [43] only has 214,000 structures). Nonetheless, creating sequences without corresponding structures may result in suboptimal outcomes [34]. Alternatively, structure-based models can offer details of the models with structural features [12], like structural paratope and epitope information, along with additional interpretable physicochemical properties [6]. Furthermore, it is generally assumed that most antigenic determinants exhibit some degree of conformational (i.e., discontinuous) structure [32]. Hence, including Ab structural information, either as an input or output, can aid in identifying potential modes of action. Nevertheless, the required Ab structure is often not available. For instance, there are only around 2000 to 5000 unique Ab–Ag complex structures [44,45], while the number of Ab sequences is over 10^13^. Therefore, a hybrid model that utilizes both sequence and structural information is considered optimal for Ab design [34].

## 2. Antibody Design

### 2.1. Role of Antibodies in Mediating Protein–Protein Interactions

PPIs play a crucial role in various cellular responses and functions, making them essential targets for the development of biomarkers and pharmaceuticals. To save experimental resources, a variety of machine learning (ML) methods have been developed, primarily focused on predicting PPI sites or residues, and these methods have been extensively reviewed [46]. Among many PPI cases, Ab–Ag interactions are distinct because most cross-interface hydrogen bonds are generated between sidechains rather than between backbones [47]. Moreover, interfaces between Ab and Ag are likely to exhibit fewer hydrophobic interactions than those observed in typical PPIs [5]. The quantity of AAs involved in the design process also differs. For instance, processes such as VDJ recombination and somatic hypermutation expand immunoglobulin (Ig) diversity to an experimentally confirmed extent greater than 10^13^ and could theoretically surpass 10^26^ [48,49]. On the other hand, the diversity of non-immune protein ranges from 10^5^ to 10^6^ [41].

Many researchers proposed that the fundamental principles governing PPI prediction can be adapted to predict Ab–Ag interactions as well [50]. An example of this is the DL-based model for the one-sided design of general PPI interfaces, which was trained with general peptide ligands and their binding complexes, then applied to Ab–Ag interfaces [33]. The highly successful attention DL-based *Binding-ddg-predictor* [15] (Supplementary Note S2) redesigned the CDR to enhance Ab affinity (toward multiple virus variants) and was validated with *SKEMPI* (Structural database of Kinetics and Energetics of Mutant Protein Interactions) version 2 [51]. This kind of approach (training with PPI, then application to Abs) is theoretically amenable with DL, as training with general 20 million protein sequences can be fine-tuned on target sequences to be optimized [52]. Additionally, general hot-spot prediction methods [53] may be applicable to immunogenic regions [54].

### 2.2. Generative Modeling for Antibody Sequences

In this section, we discuss how generative modeling techniques are used to predict and optimize Ab sequences. Gated recurrent units (GRUs), long short-term memory (LSTM) networks, variational autoencoders (VAEs), generative adversarial networks (GANs) and language models (LMs) have been utilized as deep generative models in molecular design [55,56] and computational protein design [57,58,59,60]. Language model-based sequence generation can involve the use of either an autoregressive model (e.g., *ProtGPT* [61], *ProGen* [62]) or non-autoregressive language (e.g., *AntiBERTy*, *ESM*) [63]. Consequently, it is expected that these generative DL methods have also been applied to generate or design novel Ab sequences [64]. The designs of Ab sequences have primarily focused on the CDR region, as it determines binding specificity [34]. The design of protein (including Ab) sequences refers to either sidechain design with a fixed backbone [65] or concomitant design of both the backbone and sidechain [66]. We consider generative methods for Ab sequences to contain both cases, as Jin et al. asserted [34]. Generative methods for Ab sequences include the identification of tight-binding Abs using variational Bayesian neural networks [67]. Furthermore, an ensemble approach utilizing five CNNs, along with additional methods, such as a VAE, a genetic algorithm-augmented KNN, and a genetic algorithm-augmented CNN, was employed to generate novel CDR-H3 sequences [68]. 

#### 2.2.1. Introduction to Gated Recurrent Units (GRUs) and Long Short-Term Memory (LSTM) Models

**Figure 2:**
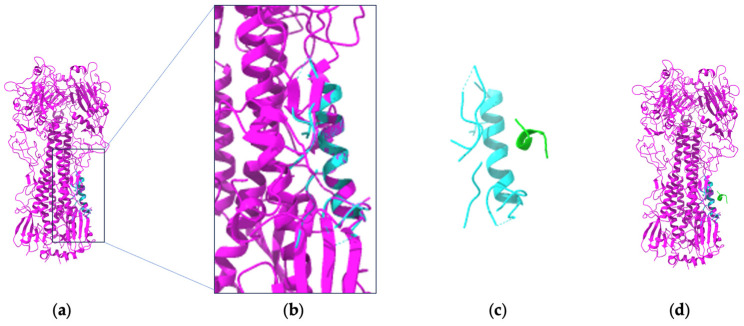
An example of *iNNterfaceDesign* running with human H3 Influenza hemagglutinin. The *iNNterfaceDesign* treats the structures of protein as the 3D object to be captured. (**a**) Target (3ztj_ABCDEF); (**b**) pocket in target; (**c**) pocket + binder; (**d**) target + binder. Target: pink; pocket: cyan; binder: green.

Both GRUs and LSTM models are variations of RNNs designed to tackle the vanishing and exploding gradient challenges inherent in traditional RNNs. For the generation of CDR-H3 sequences and synthetic Ab–Ag structures, Robert and Akbar utilized a GRU-based model [54]. Their methodology was predicated on the energetically optimal binding structures located within a 3D lattice, providing a copious volume of training data. It is worth noting that LSTM models have gained substantial popularity over GRUs within the broader DL community. A parallel trend is discernible in the field of Ab–Ag studies as well [2]. For example, LSTM memory models were used to generate novel Ab sequences for affinity maturation [39] and the classification of Ag specificity (e.g., predicting the probability of each sequence variant as either Ag binders or non-binders) [9]. Since these approaches require a massive amount of sequence data for prioritization, they utilized deep-sequenced libraries of therapeutic Abs. An LSTM was also used to generate Ag-specific CDR-H3 sequences with respect to various developability parameters [69]. Humanization of Ab sequences has been an important goal to minimize the immunogenicity of mAbs derived from xenogeneic sources and to improve effectiveness in the human immune system [70]. Therefore, LSTM models have been used to distinguish natural human Ab sequences from those originating from other species [71]. Another LSTM application of protein sequence design is *iNNterfaceDesign* [33,72]. Specifically, it designs one-sided PPI interfaces based on features of the protein receptor. Here, we show an example that we could run (Figure 2, Supplementary Note S3). *iNNterfaceDesign*-validated benchmarks include Ab–Ag complexes. Using an LSTM model with attention, it designs binder sequences (from poly glycines) with two steps (e.g., construction of binding sites centered at anchor residues, extraction of features of the binding sites, and prediction of AA sequences). Just as *DeepAb* [12] does, *iNNterfaceDesign* [33] uses the *PyRosetta* package [73]. Like other *Rosetta*-based design research [74], *iNNterfaceDesign* uses a native sequence recovery rate as the success metric. It is very encouraging to observe that *iNNterfaceDesign* achieves better sequence recovery rates than *FastDesign* [75] (*RosettaDesign* [66] during *FastRelax* [24]). Given the long history of PPI design with *Rosetta* [76], it was an expected direction that *Rosetta*-based protein interface design would incorporate DL-based modeling.

#### 2.2.2. Introduction to Variational Autoencoders (VAEs)

VAE models have been widely employed in various unsupervised DL applications. Unlike GANs, VAEs enable visualization of the latent space, allowing for the representation of similar clusters together. For Ab modeling, VAEs have been utilized to model B-cell receptor (BCR) recombination. For example, Friedensohn et al. identified sequence patterns that are predictive of antigenic exposure by VAE [77]. Later, they experimentally confirmed their binding specificity to target Ags. Another example is to learn the rules of VDJ recombination [78]. A VAE is used also to directly generate the 3D coordinates of immunoglobulins with torsion and distance awareness [79]. As shown for cryo-EM-based 3D volume generation per class [80], VAEs also prove useful in latent space sampling in Ab design.

#### 2.2.3. Application of Generative Adversarial Networks (GANs)

GANs have been used to model various properties, including images [81]. For Ab sequence design, GANs have been used to design mAbs, which retain typical human repertoire characteristics such as diversity and immunogenicity while biasing the libraries to achieve other biotherapeutic features. In particular, Just-Evotec Biologics used a Wasserstein-GAN (WGAN) with gradient penalty for this purpose [82]. To bias their GAN toward molecules with developability properties of interest, they utilized transfer learning. Like *DeepAb* and *BioPhi* [83], they used the Observed Antibody Space *(OAS)* database [84] for training and testing. This database contains more than five hundred million human sequences from more than five hundred human subjects. For Abs, it contains more than 118,386 paired heavy- and light-chain sequences and unpaired sequences.

#### 2.2.4. Introduction to Autoregressive Method

**Figure 3:**
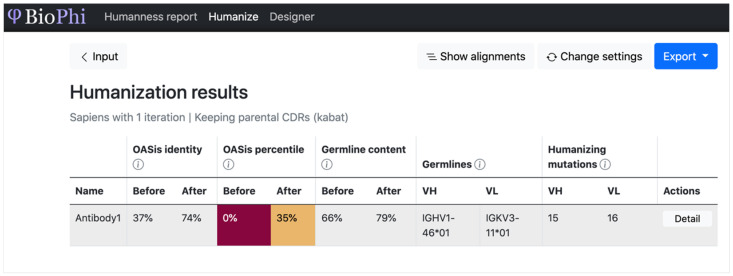
An example *BioPhi* humanization result. The *OASis* identity can be used as a threshold to separate human, humanized, chimeric, and murine Abs. The *OASis* percentile represents the percentile of *OASis* identity among therapeutic Abs (Supplementary Note S4).

Autoregressive methods in the DL field refers to models that use previous output as input, often for sequential data. One autoregressive generative method for Abs includes the co-design of sequences and 3D structures for CDRs [34], as we described in the Ab structural modeling section, since it iterates design along with updated sequence and structural information. Other examples include causal CNNs and transformers. Specifically, using a residual causal dilated CNN, Shin et al. generated millions of novel Nb sequences [60]. Recently, *BioPhi*, a platform for Ab humanization, was released [83]. It is constituted with *Sapiens*, a transformer-based Ab sequence humanization model, and *OASis*, a humanness evaluation program based on a 9-mer peptide search in the *OAS* database [84]. We found that *BioPhi* is a very user-friendly application. For example, it provides an easy-to-use website interface. Figure 3 represents a possible use case. Among more than 26 DL-based Ab modeling programs, this is one of the few cases that provides such a function. As we tried this program on our own hardware, the provided instructions for installation and execution were easy to follow as well. The runtime is very fast (i.e., fully completing within 1 min).

Most LMs use autoregressive methods, and there are several LM-based Ab sequence generation tools. For example, the Gray group shared the Ig Language Model (*IgLM*), which is trained with 558M Ab heavy- and light-chain variable sequences [85]. The *IgLM* generates full-length Ab sequences based on chain type and species of origin. By diversifying loops within an Ab, it creates high-quality synthetic libraries that exhibit biophysical properties consistent with natural Ab sequences. These synthetic libraries also demonstrate lower immunogenicity and greater resemblance to human Abs compared to baseline models. Another LM-based Ab sequence generation method is the ESM-1b transformer-based, ML-guided Antigenic Evolution Prediction (MLAEP) model [86]. *ReprogBERT* generates diverse Ab (CDR) sequences (more than two-fold increase) without losing structural integrity and naturalness [87].

## 3. Antibody Structural Modeling

### 3.1. Fragment Variable Structure and Predicting the Impacts of Mutations on the Structure and Function

**Figure 4:**
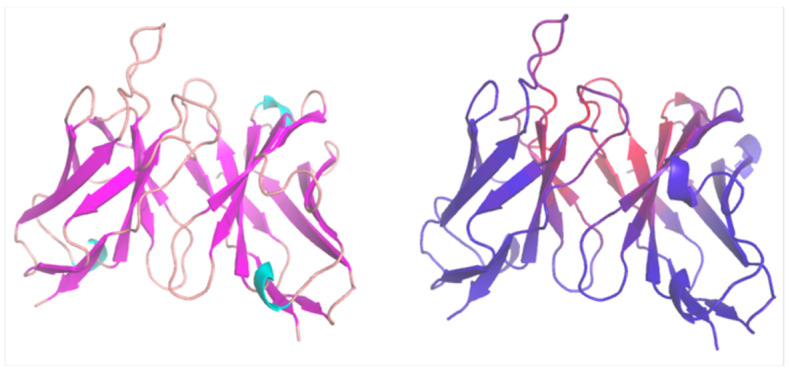
*DeepAb*-predicted Fv structures. **Left**: colored by secondary structures (i.e., loops in salmon and beta sheets in pink); **right**: colored by attention scores (red residues have high attention values, while blue residues have low attention values).

We include this subsection on fragment variables (Fvs), as they comprise the VH and VL domains of Abs, representing the smallest segment retaining the complete binding capacity of the intact Ab. The CDR region, which many computational modeling tools aim to predict, is also located within the Fv. Therefore, predicting binding based on mutations in the Fv region has become a prevalent objective. *DeepAb* is one of the recent programs addressing this pressing requirement. *DeepAb* details the alterations in categorical cross entropy of designed Ab sequences. Specifically, *DeepAb* offers predictions on the effects of Ab mutations on binding against a target, such as lysozyme. Furthermore, *DeepAb* predicts the structures of both Fvs and Nbs. Its Fv structure prediction accuracy was the best among four benchmark methods across all loops. Fv structure is predicted with two stages (i.e., identifying residue relationships and structure refinement). The first stage uses ResNet, which predicts relative distances and orientations between pairs of residues, similar to *trRosetta* [88]. Interestingly, *DeepAb* builds initial protein structures through multi-dimensional scaling (MDS) to bypass expensive sampling for much of the Ab structure. This approach becomes possible due to the high conservation of the framework structural regions of Abs. This MDS approach is different than most computational protein structure predictions [88,89] that sample protein backbone torsion angles (phi and psi) explicitly. Of course, further refinement is needed to remove clashes and non-ideal geometries right after MDS-based initial structure generation. As transformer attention visualization [90], *DeepAb* uses crisscross attention [91] to represent which residues attend more with each other (Figure 4). The second stage is a *Rosetta*-based protocol for structure realization/optimization using quasi-Newton minimization that has been used traditionally [92]. At this stage, explicit values of protein backbone dihedral angles and d_CA_ are used. As a recent dilated CNN-based embedding cluster protein function, *DeepAb* further projected sequence-averaged LSTM embedding by species and loop structures. When we tested this program, installation and execution were easy to follow (Supplementary Note S5).

**Figure 5:**
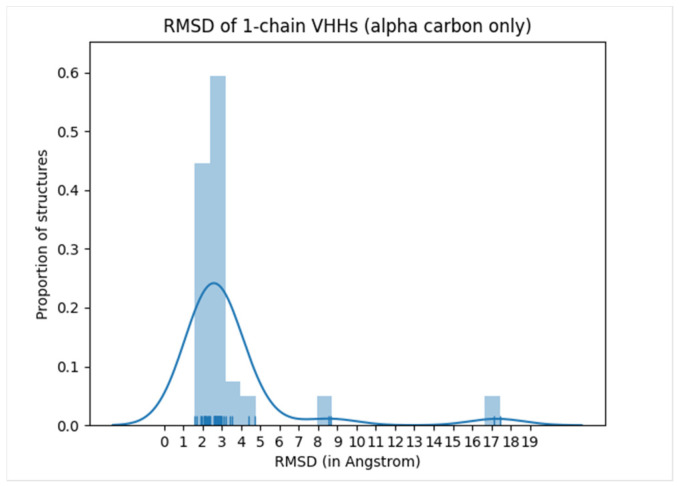
Small benchmark result of *IgFold* with Nb structures. All predictions that result in a 8~17 Å root mean squared deviation (RMSD) between experiment structure and predicted structure have floppy long terminal (either N-terminal or C-terminal) regions in experimental structures. Therefore, it is quite likely that either the experiments themselves were incomplete or *IgFold* prefers well-folded Nb structures.

Other Ab structure prediction methods using DL include *IgFold*, which is a pre-trained LM [93]. Despite not employing VAE, *IgFold* resembles *IG-VAE*, as it produces 3D coordinates of full-atom Abs directly. This direct reconstruction process is carried out by graph networks for the backbone atom coordinates and by *Rosetta* for the sidechain. As with *IgLM*, it is trained on 558M natural Ab sequences. *IgFold* predicts Ab structure faster than *DeepAb* and *AlphaFold* with comparable or slightly better accuracy. The rise of these LM-based Ab models has been expected due to the development of various LM-based protein designs [61,94]. Here, we share our independent benchmark result with *IgFold* (Figure 5, Supplementary Note S6, see Methods section for detail). Even without *PyRosetta*-based structure refinement, most predictions are accurate and produced with fast execution speed (i.e., a few seconds per ~100 AA sequence, even without a GPU). 

Another recent attention-based DL model of immune protein structure prediction is ImmuneBuilder [95]. It predicts the structure of Abs (ABodyBuilder2), Nbs (NanoBodyBuilder2), and T-cell receptors (TCRBuilder2). A notable improvement of ABodyBuilder2 is that it ran over a hundred times faster than Alphafold-Multimer [29], while it predicted the CDR-H3 loop structures with marginally better accuracy in Abs and Nbs. This is interesting, considering that ABodyBuilder2 is an Ab-specific version of the structure module in Alphafold-Multimer with several tweaks. Similar results were reported for NanoBodyBuilder2 and TCRBuilder2 as well. Errors are estimated for every residue with an ensemble of structures. 

### 3.2. Methods and Techniques in Screening for Binding Antibodies

Numerous programs have been generated to address Ab–Ag interactions. Given the vast number of Ab sequences to assess, these quick in silico screening techniques have been eagerly anticipated. *DLAB* (Deep Learning approach for AntiBody screening) [96] is one such example. *DLAB* is one of the structure-based Ab-DL methods, along with the topology-based approach [50] and the geometric representation of the surface patches [97]. It uses scores of the docking poses of Ab–Ag pairings. However, it adopts three-dimensional gridding with Conv3D. While the depiction of the protein structure/interface within a voxel grid is common in other protein DL models [98,99] and relatively easy to comprehend, it is acknowledged that this approach is computationally demanding, particularly in terms of memory usage [100]. The input size of the network restricts the voxel space to a single size [5]. In fact, most traditional multilayer perceptron (MLP) and CNN architectures do not robustly deal with rotation-invariant features [101,102]. Therefore, efforts toward SE(3) representation such as data augmentation [103], extraction of spatially invariant features as seen in spatial-VAE [104], and point cloud data [105] are desired. Trained with Ab structures (modeled with *ABodyBuilder* [106] and *ZDOCK* [107]), *DLAB* predicts Ab–Ag binding for Ags with no known Ab binders. This trait is similar to *DeepAb* [12], which shows moderate predictability of mutational tolerability, even without explicit knowledge of the Ag. Therefore, *DLAB* and *DeepAb* can be useful for early-stage development of Ab therapeutics (i.e., virtual screening).

**Figure 6:**
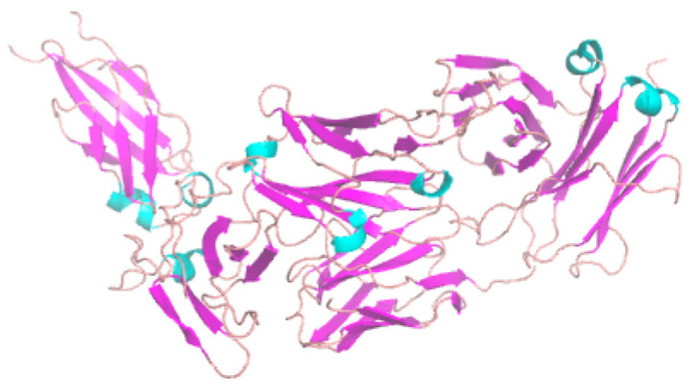
One of the *Binding-ddG-predictor*-applicable structures (i.e., beta sandwich protein). *Binding-ddG-predictor* is a semi-generative method (i.e., it generates an in silico mutation library of CDRs). Specifically, it ranks sequences by trained geometric neural network in such a way that they should improve Ab binding to the SARS-CoV-2 Delta variant RBD).

In an alternative screening approach, Kim’s team employed LM-based affinity maturation techniques [108]. They examined fewer than 20 variants for each Ab, aiming to improve binding affinities. This effort also included Abs known for their excellent thermostability and their ability to neutralize Ebola and SARS-CoV-2 pseudoviruses. A noteworthy takeaway is that more than half of the mutations proposed in this LM pertain to framework regions, areas traditionally deemed less relevant than the CDR [109]. Other LM (*ProtTrans* [110])-based prediction methods of the binding affinity of Abs (BCR) and Ags include *DeepAIR* [111]. The *Binding-ddg-predictor* [15] focuses on the design of Ab–Ag interfaces, employing an attention-based geometric neural network for analysis. Specifically, the geometric part of the model learns an embedding for each AA based on the proximity of its neighboring atoms. The attention network, reflecting the learned geometric embeddings, recognizes key residue pairs near the protein interface that have an impact on binding affinity. As a result, it generates libraries of CDR mutations, ranking each mutation according to its influence on binding affinity and structural stability. After this cyclical optimization process, this DL approach improved an Ab, which displayed significantly increased and more effective virus-neutralizing activity compared to the original Ab. We present an example that can be leveraged using the *Binding-ddG-predictor* (Figure 6). In this structure, even a single mutation (among 628 total AAs) was predicted to alter the ddG by 0.3.

### 3.3. Strategies in Designing Both Sequence and Structure in Optimizing Antibody Efficacy

A recent trend in Ab modeling includes the co-design of sequence and structure to overcome previous approaches. For example, Jin et al. employed an autoregressive/generative graph neural network (GNN) to co-design the sequence and 3D structure of CDRs [34]. Essentially, this approach is similar to *RosettaAntibodyDesign* [112], as it focuses solely on predicting CDRs while leaving the framework region unaltered. However, instead of a physics-based score function as used in *RosettaAntibodyDesign*, they used graph-based DL. Here, a graph is a sequence–structure pair, and it models the conditional relationship between a CDR and its environments. This unique capability is possible by modeling protein backbone angles on top of AA identity and a joint graph representation that connects between CDR residues and between the CDR and framework (rather than single-residue prediction with CDR only). Since framework+CDR has more than 100 residues, graph convolution over the whole graph (>100 nodes) is challenging. Therefore, they clustered framework residue into K-mers to minimize the graph size. Unlike previous autoregressive models that never update residue distances, even when a new residue is added, their model updates residue distances whenever a new residue is introduced. Consequently, this method was very effective with a rotation/translation-invariant loss function and outperformed all other methods (e.g., *RosettaAntibodyDesign*, LSTM, and AutoRegression) in terms of speed, sequence recovery rate (<29% vs. 34%), and structure prediction. As a multi-objective optimization, this model designs CDR-H3 sequences that have higher neutralization probability as well.

### 3.4. Application of Diffusion Methods

Another co-design of CDR sequence and structure involves a diffusion probabilistic model, which demonstrated competitive binding affinities according to *Rosetta* energy functions, other protein design metrics, and in vitro experimental validation [113]. *AbDiffuser*, in particular, utilizes the Aligned Protein Mixer (APMixer), an innovative neural network designed for the processing of proteins from an aligned protein family [113]. APMixer is more memory-efficient and operates more rapidly than GNNs and transformer-based models. Additional applications of diffusion models in Ab applications encompass *diffusioN Optimized Sampling* (*NOS*) [63] and *EvoDiff* [114]. *NOS* enables direct execution of designs within the sequence space, effectively bypassing substantial constraints associated with structure-based methods (i.e., limited data and complex inverse design challenges). Remarkably, *NOS* shows better sequence recovery by infilling than *IgLM* [85], *RFdiffusion* [115], and *DiffAb* [116]. *EvoDiff* utilizes evolutionary data to generate proteins beyond the reach of structure-based modeling techniques. It improves Fréchet ProtT5 distance (FPD) even better than *RosettaFoldDiffusion (RFdiffusion)* [115]. The denoising diffusion probabilistic model has recently been employed in numerous general protein structure prediction and design models [115,117,118,119,120,121,122,123] and in generating end-to-end protein–ligand complexes [124]. Hence, the application of the SE(3) diffusion model specifically to Abs was anticipated. A drawback of the diffusion-based Ab model is its need for an Ab framework attached to the target Ag.

### 3.5. Graph-Based Supervised Learning for Biophysical Property Prediction

In this section, we discuss the application of graph-based learning in predicting biophysical properties of Abs. GNNs have been widely used across all scientific domains [17,125]. For applications in property prediction, message-passing neural networks (MPNNs) have been used to predict IC_50_ values with anti-SARS database and molecular property [125]. For PPI research, GNNs have been used to predict various features, such as the 3D structure of a protein–protein complex [126], synergy scores of drug combinations [127], and effects of mutations on protein-protein binding affinity [128], PPI link [129], PPI site [130,131] and patterns in protein–protein interfaces [132]. For Ab modeling specifically, GNNs have been used to co-design the sequence and 3D structure of CDRs and affinity maturation [34,133].

To consider physiological impact of novel therapeutics, DL-based Ab developability filtering methods [9,82] can save a huge amounts of resources. These Ab developability filtering methods aim to predict various biophysical properties. These physicochemical/biophysical properties include thermal and colloidal stability, aggregation, fragmentation, hydrophobic patches/surfaces, solubility, post-translation modification (PTM), and half-life (pharmacokinetics), as reviewed in [1]. Most of these properties can be trained on either numerical or categorical values with DL methods. However, when Ab-related biophysical predictions use generative methods, the copy problem (i.e., generative modeling may reproduce the training data too closely) [134] should be avoided to generate reasonably novel Ab sequences. Overall, computational constraints that govern the developability of therapeutic Abs are summarized as evenly distributed hydrophobic residues across the surface, avoiding glycosylation motifs and CDR residues with reasonable levels of charges [135]. Various non-DL-based in silico methods for Ab developability parameter computation were summarized by Akbar et al. [41]. Other aspects of Ab developability, such as avoiding unusual CDR sequences that are not explicitly explained by biophysical properties, can be examined through the perplexity calculated from an ensemble of LMs [136]. A supervised graph-based approach shows considerable potential in predicting biophysical properties during Ab design. Even when non-DL ML methods such as random forest, Gaussian processes, and nearest neighbors or a simple MLP were employed, graph-based signatures demonstrated their effectiveness in capturing the interaction interfaces between Ab and Ag and in predicting binding affinity [137].

### 3.6. Curation of Sequence and Structural Datasets to Develop Unsupervised Machine Learning Methods

To achieve success with unsupervised ML techniques, it is essential to have high-quality datasets comprising sequences and structures. With abundant data, these methods can excel in identifying patterns or tendencies and grouping them within the latent space, broadening their domain of application. Moreover, it is crucial to tackle data bias in the training set to guarantee precise extrapolation. However, the existing databases of Ab and PPI sequences and structures (Table 1 and Table 2), which were used for development of various DL-driven Ab modeling programs (Table 3), do not offer comprehensive coverage of all known antibody sequences. Additionally, many Ab sequences and structures are redundant. This obvious limitation of the datasets is evident, given the fact that in addition to the core regions of Abs (i.e., the framework) but the CDR region alone requires a 20^60^ possible combinatorial search space of various sequences [34]. Therefore, to minimize data bias, reasonable in silico generation methods (such as *Absolut*! [54]) can be considered to reduce the gap. Additionally, interpretable models would be useful to assess data completeness [138]. Effective embedding can also minimize the data bias issue by better intra/extrapolation. For example, recently, a dilated CNN-based embedding was employed to model a protein function [18]. Specifically, the findings demonstrate that using contextualized word-embedding representation for protein sequences [139] eliminates the need to incorporate explicit structural information, which, in turn, effectively simplifies the modeling process [140]. These computational efforts will eventually allow for effective Ab sequence modeling. A similar analogy can be found in computational protein structure prediction. Due to recent advancements in multiple sequence alignment (MSA)-based structure prediction [141], the sampling process has become significantly more efficient. This enhancement significantly addresses the vast potential inherent in protein folding, a concept previously referred to as Levinthal’s paradox [142]. Therefore, as the Akbar group has contributed a substantial amount of Ab structure data for training with GRUs [54], scientists have widely adopted transformer based MSA programs like *AlphaFold* [141] and *RoseTTAFold* [143] to generate synthetic Ab structures. Nonetheless, it is crucial to acknowledge the superiority of LMs, such as *IgFold* [93] and *OmegaFold* [144], in terms of the overall prediction accuracy for Ab structures. This holds true particularly for the complex structure of CDR3. Furthermore, these LMs exhibit a notable advantage in terms of speed when compared to *AlphaFold*, representing a significant improvement in the field.

## 4. The Role of Antibodies and Deep Learning in the Fight against SARS-CoV-2

### 4.1. Understanding How SARS-CoV-2 Interacts with Host Cells

To more effectively illustrate the application of DL in Ab research, we discuss SARS-CoV-2 in this section. The human death toll of SARS-CoV-2 is estimated to be 10~20 million so far [150]. Additionally, this disease has impacted economics negatively. Therefore, many structural studies about the disease have been reported, such as those investigating Nb [151] or Fab [152] binding to the receptor-binding domain (RBD). Often, structural studies of the target protein suggest plausible routes of development, such as the suggestion of an anti-neuraminidase Ab as a starting point for the design of an anti-SARS Ab [153]. The authors of the abovementioned NB and Fab binding studies attempted to inhibit angiotensin-converting enzyme 2 (ACE2) binding either directly or indirectly. This ACE2-RBD interaction can be blocked by fusion proteins (such as extracellular portions of ACE2 or RBD fused to the Fc portion of human IgG1) as well [154]. Fusion glycoproteins lie on the surface of enveloped viruses and are important for the cell entry of viruses [155]. This fusion protein-targeting approach has been gaining traction for other viruses, such as respiratory syncytial virus. For example, the King group computationally designed immunogens that induced potent neutralizing Ab responses [156]. All three classes of fusion proteins (i.e., I, II, and III) have different structures, mechanisms (triggering molecule/pH or reversibility between pre-fusion and post-fusion), and applicable viral families. For SARS-CoV-2, which belongs to the Coronaviridae family, class I fusion protein acts as the fusion machinery. As a result, class I fusion proteins have been studied most extensively. To develop ML models to combat SARS-CoV-2, it is necessary to have a specific dataset for class I fusion proteins. Well-known cryo-EM structures show that class I fusion proteins have a high proportion of alpha helices in their post-fusion conformation with coiled coils [152]. The structural stability of the pre-fusion and pre-hairpin states, which are intermediate stages between pre-fusion and post-fusion, is lower compared to that of the post-fusion state [141]. This implies that the examination of pre-states should incorporate both kinetic and thermodynamic properties, leveraging tools like molecular dynamics or Monte Carlo simulations, as well as quantum mechanical calculations. In contrast, the post-fusion state can primarily be evaluated based on its thermodynamic properties. Stabilization of the pre-fusion and intermediate states prior to reaching the post-fusion stage can aid in averting viral infections. Alternatively, as suggested earlier, direct inhibition of the binding interaction between ACE2 and the receptor-binding domain (RBD) is also a viable strategy.

### 4.2. Overview of Experimental Datasets in Studying SARS-CoV-2

We present various experimental datasets that were used as training sets for DL related to SARS-CoV-2. Neutralizing antibodies (nAbs) are effective for the prevention and treatment of SARS-CoV-2-related infections. Therefore, phage-display immune libraries were employed to isolate effective nAbs against SARS-CoV-2 from pooled peripheral blood mononuclear cells (PBMCs) of COVID-19 convalescent patients [157]. This phage-display screening identified a neutralizing IgG that attaches to an epitope located on the N-terminal domain of SARS-CoV-2 [158]. mAbs that neutralize and obstruct the binding of the SARS-CoV-2 spike protein to ACE2 can also be discovered through target-ligand blocking methods and BCR sequencing. This involves linking the BCR to Ag specificity via sequencing [159]. For such campaigns or screenings, the dissociation constant (K_D_) and IC_50_ are crucial measurements. The binding kinetics (K_D_) of the mAbs to the target molecule are typically assessed using surface plasmon resonance (SPR) [1]. To quickly assess developability, protein thermal unfolding temperatures, such as the midpoint temperature (T_m_) and onset temperature (T_onset_), can be gauged using differential scanning fluorimetry. Of course, these experimental screenings can be lessened or supplemented by virtual screenings of Abs [9,39,96].

### 4.3. How Deep Learning Is Advancing Research on SARS-CoV-2

In this section, we present a range of DL techniques used to address SARS-CoV-2 challenges on top of a well-summarized review [160]. For instance, CNNs have been employed to pinpoint the representative genomic sequence of SARS-CoV-2 among various viral genome strains [161]. Furthermore, DL methods have been utilized to repurpose existing drugs for COVID-19 through network-based approaches [162] and to analyze COVID-19 computed tomography imaging using UNET [163]. Thus, the impact of DL methods in the fight against SARS-CoV-2 is significant, with an ever-growing list of specific contributions. However, DL’s role in SARS-CoV-2 Ab design has largely been confined to predicting binding affinity related to general PPIs [164]. This limitation stems from the challenge of fine tuning existing general PPI-based DL models for SARS-CoV-2-targeted Ab design due to a scarcity of SARS-CoV-2-specific datasets. For instance, only around 500 experimentally determined SARS-CoV-2-specific structures exist (Table 1). Nevertheless, given the recent successful applications of DL for the optimization of specific SARS-CoV-2 variants [15], it is expected that the development of DL-based COVID-19 Ab designs will become more prevalent. DL methods have expedited the development of therapeutics by identifying epitopes, offering a time-efficient alternative to experimental screening [165,166]. Many of these methods have been employed to combat SARS-CoV-2 or hold the potential to be applied for the same. Specifically, T-cell epitope prediction utilizing artificial neural networks includes RNN-based prediction of peptide–human leukocyte antigen (HLA) class II binding [167], along with sparse encoding and BLOSUM [168] encoding-based prediction of HLA-DR binding, an MHC class II cell surface receptor encoded by the HLA [169]. Conversely, B-cell epitope prediction employing artificial neural networks includes RNN-based prediction of linear or continuous B-cell epitopes of an antigen [170].

There is substantial room for enhancements in DL methodologies used in Ab design that can be universally applied, not only for SARS-CoV-2. First, incorporating evolutionarily conserved sequence information (MSA) can enhance DL-based Ag design, as its effectiveness has already been demonstrated in general protein structure prediction [141,143] and design [74]. Aside from the CDR-H3 region, Ab sequences exhibit high similarity, which supports the feasibility of using MSA. Secondly, diversifying Ab repertoires reflecting in vivo insertion and deletion of AAs into the V region, post-translational modifications, and the use of non-protein cofactors [171] have not been fully realized in DL approaches [41]. Nonetheless, this limitation is being addressed; for instance, glycan information in proteins has been converted to lattice representations to generate a wealth of DL training data [54]. DL has not advanced as much in certain areas, including germ lines, Ab formats (Fc-fusion, scFv, and Fab), specific sequence liabilities (deamidation and glycosylation sites), and clearance likelihood [4]. Lastly, many generative programs for Ab sequence design lack appropriate code/document sharing. To make a greater impact on the community, more collaborative approaches should be encouraged.

Establishing a foundation for ML and structural modeling for SARS-CoV-2 requires careful consideration. For instance, smaller monomer–monomer interactions (such as those between the RBD and Nbs) can be investigated via docking [29,172]. However, the entirety of the class I fusion protein often exceeds 2000 AA, making *AlphaFold-Multimer* studies impractical due to memory limitations. Attempts to circumvent this by removing the C-terminal region to fit into the *AlphaFold-Multimer*’s memory (such as on Google Colab Pro+) often results in orientations between chains that significantly deviate from experimental findings. As such, a threading method with individual structure is recommended. Vaccine design strategies using non-DL ML methods encompass combinatorial ML approaches such as support vector machine, k-nearest neighbors, logistic regression, random forest, and extreme gradient boosting. These methods underpin reverse vaccinology, which starts by predicting the optimal vaccine candidate through bioinformatics analysis of the Ag genome [173].

## 5. Conclusions and Future Directions

We have highlighted several DL-based Ab modeling programs [12,15,33,79] that utilize *Rosetta* [174] for ddG calculation, structure idealization, sequence design, sidechain optimization, and visualization. Given that the *Rosetta* group is one of the forefront groups of computational protein design [66], it has been expected that they will continue incorporating DL methods to enhance their current capabilities in Ab design [175,176] and Ab and protein stability improvement [177,178]. This will aid in addressing challenges such as Ab thermal stability [179] and Ab structure prediction [180].

Reliable Ab–Ag interaction prediction, particularly due to the diverse conformations of the H3 loop in CDR, has remained an elusive goal [12,68]. For example, *AlphaFold-Multimer* has not demonstrated reliable Ab–Ag binding prediction [29]. The limited amount of data, varying biological platforms [4], and structural alterations upon Ag interaction [181] also present challenges when applying DL to Ab research. Consequently, it has been a typical approach to employ databases that encompass both Ab-specific and broader PPI data [182], as we previously mentioned.

However, recent advances in DL approaches for Ab sequence design and classification have accelerated Ab development, enabling the exploration of a much larger protein sequence space than display libraries can offer [9]. For instance, generating a vast number of simulated 3D Ab–Ag complex structures that represent various biological complexities [54] may provide a valuable foundation for the enhancement of DL techniques in Ab sequence design. Furthermore, LM-based Ab structure prediction methods such as *IgFold* and *OmegaFold*, which are faster and offer similar or better quality predictions compared to *AlphaFold*, are starting to emerge. LMs hold considerable promise and are highly generalizable. For instance, Hie and colleagues have shown that their approach is not limited to Abs but can be effectively extended to other proteins as well [109]. In every test instance, it was observed that the general-purpose protein LM yielded better results than methods focused solely on Abs.

Here, we share opinions that are applicable both to general PPIs and Ab sequence design. First, other than two cases [35,83], most current sequence-based DL models for Ab sequence design and modeling represent protein sequences with one-hot encoding. This traditional method often used in RNNs is fine for most cases and sometimes ideal for ease of training on the available limited data [12]. However, transformer models can deal with long protein sequence information more easily [19]. Therefore, it better captures relationships between sequences with an attention model and can visualize these relationships intuitively as well [31].

Secondly, it is worthwhile to focus on updating models originally designed for individual monomer prediction. A prime example of this methodology is *AlphaFold-Multimer* [29]. It employs the same *Evoformer*, a transformer with multiple sequence alignment (MSA) representation and pair-wise information, as the original *AlphaFold* [141]. However, it adopts a longer-distance cutoff for the frame-aligned point error (FAPE) loss. This loss refers to the distances between the actual and predicted atoms in the local reference frame of each residue. This adjustment is implemented to facilitate the training of interchain pairs. Notably, this method has demonstrated higher accuracy compared to previously prominent techniques, including *AlphaFold*-refined *ClusPro* docking. Thirdly, the protein–protein docking score is better in a standardized form between the DockQ score and TRScore [183] for consistent comparison and reporting.

Finally, as highlighted by Shaver et al. [4], the use of pre-training techniques like masked language modeling (MLM) embodied in models such as Bidirectional Encoder Representations from Transformers (BERT) [184] and the Generative Pre-trained Transformer (GPT) [185] could greatly benefit the Ab sequence research community. The MLM technique involves obscuring certain residues in a protein sequence and training the model to predict the hidden AAs based on the rest of the sequence. This approach has already demonstrated potential in protein sequence applications [19,186]. One of its key benefits is its swift deployment time. Even though the training process and final structural refinement can be time-consuming, the actual implementation of pre-trained models in both the pharmaceutical industry and academic research is quite manageable. As a result, we can anticipate the development of more LM-based Ig modeling methods, such as *IgLM* and *IgFold*.

## 6. Methods

We used *ChimeraX* [187] to generate Figure 1. The examples that we ran and the analysis scripts that we used for Ab programs are presented in Supplementary Notes S2–S6. For the *IgFold* benchmark (Figure 3, Supplementary Note S6), we downloaded the entire 1252 VHH (i.e., Nb) PDB structures from SAbDab (as of February of 2023). Then, according to the Chothia naming convention, we chose all one-chain PDB structures and removed entries with duplicated structures, leaving 51 unique representative Nb structures. We converted these into fasta files and ran *IgFold*. RMSD values were calculated by *BioPython* [188] after superimposing comparing structures. The RMSD values calculated in this way match to those calculated by *ChimeraX*.

## Figures and Tables

**Table 1:** Ab sequence and structure databases. We present mostly large-scale databases. Other databases were reviewed by Akbar et al. [41] and Wilman et al. [2].

Data Source	Description	Number of Entries
AbDb [45]	Expert-curated Ab structure database	~2 k full structures
Absolut! [54]	In silico generated Ab–Ag bindings	159 antigens times 6.9 million CDR-H3 murine sequences
AntiBodies Chemically Defined Database (ABCD) [145]	Manually curated depository of sequenced Abs	23 k sequenced Abs against 4 k Ags
CoV-AbDab (in SAbDab) [146]	Coronavirus-binding Ab sequences and structures	4 k homology models and 500 PDB structures
iReceptor [147]	Ab/B-cell and T-cell receptor repertoire data	>5 B
Observed Antibody Space (OAS) [84]	Paired and unpaired (VH/VL) Ab sequences	>1 B
SAbDab [44]	Ab structures available in PDB	>5 k

**Table 2:** PPI sequence and structure databases. We present mostly large-scale protein interaction databases. Other databases were reviewed by Akbar et al. [41]. SKEMPI V2.0 is for general PPIs, yet it is also the largest Ab–Ag binding affinity database.

Data Source	Description	Number of Entries
IntAct [148]	Binary interactions from the literature and user submissions	>1 M
MINT (in IntAct)	Protein interaction information disseminated in the literature	>130 k
SKEMPI V2.0	Structural Kinetic and Energetic database of Mutant Protein Interactions	7 k
STRING [149]	Direct (physical) and indirect (functional) PPIs	>20 B

**Table 3:** Some of the prominent antibody modeling programs. A full list of programs is presented in Supplementary Note S7.

Model	Goal	Input Type	Output	Architecture	Metrics	Note
Binding-ddg-predictor	Redesign the CDR to enhance Ab affinity (targeting multiple virus variants)	Sequence	Predicted binding affinity	Attention-based geometric neural network	kD (dissociation constant)	Through an iterative optimization procedure, this DL method found that the optimized Ab exhibited broader and much more potent neutralizing activity compared to the original Ab
BioPhi	Humanize the sequence and evaluate the humanness of the sequence	Sequence	Sequence and predicted humanization	Transformer	Accuracy (%), ROC, AUC, and R^2^	Different methods were more successful in different cases, further encouraging the assembly of a diverse arsenal of humanization methods
DeepAb	Predict the Ab mutation effect on binding	Sequence	Structure and predicted affinity	RNN for sequence representation and ResNet to predict six distances and angles	Orientational coordinate distance and AUC	Provides an attention layer to interpret the features contributing to its predictions
IgFold	Predict Ab (Fv) structure	Sequence	Predicted Ab structures	Pre-trained language model followed by graph networks that directly predict backbone atom coordinates	Orientational coordinate distance and RMSD	Representations from IgFold may be useful as features for ML models
IG-VAE	Directly generate 3D coordinates of full-atom Abs	Known IG structures	Diversified IG structures	VAE	Distance matrix reconstruction and torsion angle inference	Intended for use with existing protein design suites such as Rosetta
iNNterfaceDesign	One-sided design of protein–protein interfaces	Both sequence and structure (features of protein receptors)	Redesigned protein interface sequence and structures	LSTM with attention	Recovery rates of the native sequence and hot spot	First neural network model for prediction of amino acid sequences for peptides involved into interchain interactions
RefineGNN	Co-design of the sequence and 3D structure of CDRs as graph	Both sequence and structure	Both sequence and structure	Autoregressive/generative graph neural network	Perplexity of sequences and the RMSD	Co-designs the sequence and 3D structure of CDRs as a graph

## Data Availability

For the benchmark input and output, refer to 10.5281/zenodo.10632946. We obtained 1252 VHH (also known as Nb) PDB structures from SAbDab in 2023. We did not generate any new experimental data.

## Supplementary Materials

The following supporting information can be downloaded at: https://www.mdpi.com/article/10.3390/bioengineering11020185/s1, References [189,190,191,192] are cited in the Supplementary Materials, Supplementary Note S1. Running and interpretation of *AlphaFold-Multimer* result; Supplementary Note S2. Example *binding-ddg-predictor* script; Supplementary Note S3. Example *iNNterfaceDesign* script; Supplementary Note S4. Example *BioPhi* script; Supplementary Note S5. Running *DeepAb;* Supplementary Note S6. Example *IgFold* script; Supplementary Note S7. Detailed list of Ab modeling programs.
