# Supplementary material for: Leveraging Artificial Intelligence to Expedite Antibody Design and Enhance Antibody–Antigen Interactions
Source: Bioengineering (Basel). 2024 Feb 15;11(2):185. doi: 10.3390/bioengineering11020185 (PMC10886287; doi:10.3390/bioengineering11020185)
Supplement: Supplementary file 1 [file bioengineering-11-00185-s001.zip › Supplementary Information.pdf]

Supplementary Information

Leveraging Artificial Intelligence to Expedite Antibody Design and Antibody-Antigen Interactions

Doo Nam Kim, Andrew D. McNaughton, Neeraj Kumar*

Computational Biology Group, Biological Science Division, Pacific Northwest National Laboratory, 902 Battelle Blvd, Richland, WA, 99352, United States

* Corresponding author: neeraj.kumar@pnnl.gov; Tel.: +1-509-372-6422

Supplementary note S1. Running and interpretation of *AlphaFold-Multimer* result

Supplementary note S2. Example *binding-ddg-predictor* script

Supplementary note S3. Example *iNNterfaceDesign* script

Supplementary note S4. Example *BioPhi* script

Supplementary note S5. Running *DeepAb*

Supplementary note S6. Example *IgFold* script

Supplementary note S7. Detailed list of Ab modeling programs

Supplementary note S1. Running and interpretation of *AlphaFold-Multimer* result

Protein sequence information and desired number of models are all inputs required.

AlphaFold-Multimer reports reliability of overall *AlphaFold-Multimer* prediction (i.e., ipTM + pTM, the sum of 'binding interface prediction' and 'monomer structure prediction').

model confidence = 0.8 · ipTM + 0.2 · pTM

pTM (predicted template modeling score) is a measure of the error for the predicted structure in 3D. It ranges from 0 (wort) to 1 (best). Predictions with pTM < 0.2 are either stochastically assigned residue patterns, with negligible or no correlation with the supposed native structure or are intrinsically disordered proteins. A pTM value exceeding 0.5 is generally considered sufficiently robust to enable reliable inferences[1].

ipTM is pTM of interface. Higher than 0.75 ~ 0.85 is considered a reliable prediction [2 3].

Supplementary note S2. Example *binding-ddg-predictor* script

Example running environment:

```
run -t 10 --pty -n 1 -p dlv -A i3t-acd -u /bin/bash
srun: job 651309 queued and waiting for resources
srun: job 651309 has been allocated resources
```

Example running script that we used:

```
python scripts/predict.py data/example_wt.pdb data/example_mut.pdb
```

Example output:

```
Predicted ddG: -0.30
```

Supplementary note S3. Example *iNNterfaceDesign* script

Example *PepBB* running script that we used:

```
run_this_py="../iNNterfaceDesign_scripts/1.preprocessing.py"
input_file="PepBB.input"
python $run_this_py $input_file #took 2~5 minutes

run_this_py="../iNNterfaceDesign_scripts/2.binders.py"
input_file="PepBB.input"
python $run_this_py $input_file #took < 1 min

run_this_py="../iNNterfaceDesign_scripts/3.preprocessing_seq.py"
input_file="PepBB.input"
python $run_this_py $input_file #took < 1 min

run_this_py="../iNNterfaceDesign_scripts/4.amn_sampling.py"
input_file="PepBB.input"
python $run_this_py $input_file
```

Example *PepSep* running script:

```
run_this_py="../iNNterfaceDesign_scripts/1.preprocessing_sp.py"
input_file="input_pepsep6_long_frag"
python $run_this_py $input_file

run_this_py="../iNNterfaceDesign_scripts/4.amn_sampling.py"
input_file="input_pepsep6_long_frag"
python $run_this_py $input_file
```

Supplementary note S4. Example of *BioPhi* script

Example script that we used:

```
OASis_FILE="/rcfs/projects/i3t-acd/ML-ACD/ab/db/OASis_9mers_v1.db"

# Run full humanization & humanness evaluation pipeline
biophi sapiens mabs.fa \
   --oasis-db $OASis_FILE
   --output humanized/

# Get the Sapiens probability matrix (score of each residue at each position)
biophi sapiens mabs.fa --scores-only --output scores.csv

# Get mean Sapiens score (one score for each sequence)
biophi sapiens mabs.fa --mean-score-only --output scores.csv

# Get OASis humanness evaluation
biophi oasis mabs.fa \
   --oasis-db $OASis_FILE
   --output oasis.xlsx
```

Supplementary note S5. Running *DeepAb*

Example running script that we used:

```
run_this_py="/people/kimd999/ML-ACD/ab/code/official/DeepAb/predict.py"
Fv_seq="/people/kimd999/ML-ACD/ab/code/official/DeepAb/data/sample_files/4h0h.fasta"
python $run_this_py $Fv_seq --decoys 5 –renumber
```

Running times were reasonable given with time consuming *Rosetta* based structure refinement (e.g. Fv structure prediction took 17~50 minutes for L chain only and H and L chains, residue attention annotation and design score calculation took 1~5 minutes).

Attention can be visualized by *PyMOL*[4].

Example *PyMol* command script for this analysis that we used:

```
spectrum b, blue_red, minimum=0.1, maximum=1.3
```

Supplementary note S6. Example *IgFold* script

Example script that we used:

```
from igfold import IgFoldRunner

sequences = {

    "A":
"VQLQESGGGLVQAGGSLRLSCAASGRTGSTYDMGWFRQAPGKERESVAAINDSARTY
YASSVRGRFTISRDNAKKTVYLQMKPEDTAVYTCGAGEGGTWDSWGQGTQVTVSS",

}

pred_pdb = "1hcv_predicted.pdb"

igfold = IgFoldRunner()

igfold.fold(

    pred_pdb, # Output PDB file

    sequences=sequences, # Nanobody sequence

    do_refine=False, # Refine the antibody structure with PyRosetta

    do_renum=True, # Renumber predicted antibody structure (Chothia)

#    use_abnum=True,

)
```

Supplementary note S7. Detailed list of Ab modeling programs

Out of 26 Ab modeling programs, 16 programs mention that they generate Ab sequences. However, 4 programs are semi-generative methods (e.g., rank/screens based on binding affinity. These will work as 'direct' sequence generation method eventually. However, screening 20^n will take too long time), 1 program generates *in silico* data that needs validation, 4 programs did not share code, and 3 programs cannot be ran due to lack of consistent documentation. *BioPhi*[5] and *iNNterfaceDesign*[6] are well-documented to run at the time of this writing.

Refer an excel file (i.e., Ab_modeling_programs.xlsx) for further detail. Please be advised that we have not tested all these programs. Therefore, we could not validate all claims of authors. However, we believe that peer reviewers, journal editors and users already validated at least feasibility.

Reference

1. Temitope Sobodu. How to Deploy and Interpret AlphaFold2 with Minimal Compute. *https://towardsdatascience.com/how-to-deploy-and-interpret-alphafold2-with-minimal-compute-9bf75942c6d7* (2023).
2. Yin, R., Feng, B. Y., Varshney, A. & Pierce, B. G. Benchmarking AlphaFold for protein complex modeling reveals accuracy determinants. *Protein Science* **31**, e4379 (2022).
3. O'Reilly, F. J. *et al.* Protein complexes in cells by AI-assisted structural proteomics. *Mol Syst Biol* **19**, e11544 (2023).
4. The PyMOL Molecular Graphics System, Version 1.8 Schrödinger, LLC.
5. Prihoda, D. *et al.* BioPhi: A platform for antibody design, humanization, and humanness evaluation based on natural antibody repertoires and deep learning. *MAbs* **14**, 2020203 (2022).
6. Syrlybaeva, R. & Strauch, E.-M. Deep learning of Protein Sequence Design of Protein-protein Interactions. *bioRxiv* 2022.01.28.478262 (2022) doi:10.1101/2022.01.28.478262.
